# A Single-Institution Audit of Chemo-PORT Placements During Breast Surgery with Literature Review for Management of Common Complications

**DOI:** 10.1007/s13193-025-02456-9

**Published:** 2025-11-17

**Authors:** Ankita Nirpharake, Shalaka Joshi, Bhavika Kothari, Dileep Hoysal, Mitchelle Engineer, Jessicka Shah, Nitin Shetty, Suyash Kulkarni

**Affiliations:** 1 Department of Surgical Oncology, Breast Services, Tata Memorial Hospital and Home Bhabha National Institute, Mumbai, India; 2 Department of Radiodiagnosis and Interventional Radiology, Tata Memorial Hospital and Home Bhabha National Institute, Mumbai, India

**Keywords:** Chemo-PORT, Breast surgery, Complications management

## Abstract

Most breast cancer patients need chemotherapy for a duration of 3–12 months. The morbidity of frequent intravenous access, mainly on the contralateral upper limb is troublesome and chemo-PORT placement is helpful. We analysed a prospective database of patients who had concurrent chemo-PORT placement with breast surgery between January-2019 and December-2023 at our centre for their complication rate. Clinico-pathological data was collected from electronic medical records and analysed in SPSS-V25. A total of 408 breast cancer patients underwent chemo-PORT placement. The median age of cohort was 50 years(18–78). Breast conservation surgery was done in 201(49.3%), 207(51.7%) had mastectomy. Of 408, 195(47.8%) patients had per primum surgery, 126(30.9%), 26(6.4%) and 61(15%) were operated post-1st line chemotherapy, in the recurrent setting and for second breast primary respectively. 260(63.7%) were HER2 positive, 73 ER/PgR + (17.9%) and 75(18.4%) TNBC. Total 256(62.7%) patients received anti-HER2 therapy. The overall complication rate was 12.5%(51/408). Procedural/immediate complications were seen in 3/408(0.7%) patients which included pericardial placement, pleural cavity placement with haemothorax, and intra-operative guidewire migration in 1 patient each. Post-procedural complications were further sub-classified- as short-term complications that included PORT site hematomas in 3/408(0.7%), pocket-site infections in 4/408(0.9%); and long-term complications that included catheter-related bloodstream infections in 13/408(3.2%), catheter related thrombosis in 7/408(1.7%), persistent withdrawal occlusion in 14/408(3.4%), and catheter displacement in 7/408(1.7%).Chemo-PORT placement is an efficient and safe way of administering chemotherapy in breast cancer patients with < 1% serious complication rate. However, timely identification of serious complications can prevent a catastrophe.

## Background

Breast cancer is the most common cancers among women [1]. Almost all patients receive chemotherapy, in non-metastatic setting, it lasts for 3–18 months [2]. Vascular access remains an important aspect, a traditional peripheral intravenous line being common. A central vascular access such as a peripherally inserted central catheter(PICC) or a surgically inserted central line, is often helpful. Repeated thrombophlebitis due to intravenous access lines on the contralateral upper extremity are troublesome post-operatively, especially in breast cancer patients when the ipsilateral upper limb is at risk of lymphoedema [3]. With various vascular access devices, intravenous administration of chemotherapy has been facilitated with improvement in patients’ quality of life [4].

Chemo-PORT is safe and effective option for long-term venous access in cancer patients undergoing chemotherapy [5]. The PORT system can be used for infusion of medications, chemotherapeutics, IV fluids, parenteral nutrition solutions, blood products, and withdrawal of blood samples. Chemo-PORTs reduce patient anxiety associated with repeated venepuncture, have less interference with daily activities, reduced risk of infection and are cosmetically acceptable since placed subcutaneously [6]. There are two types of catheter systems open and Groshong’s (valved), which are available with same type of chemo-PORT. Groshong’s catheter has a valve at the tip. The opening of the valve is dependent on the application of either negative or positive pressure. In resting conditions, the valve remains closed and there is theoretically less chance of formation of thrombus and hence fewer thrombotic complications. However, it has been demonstrated that the use of Groshong’s catheter is not necessarily superior to an open catheter system in terms of early and late complications [7].

Understanding the outcomes and complications associated with chemo-PORT placement is crucial for improving patient care and safety. We have been routinely using chemo-PORTs for breast cancer patients along with primary surgery or independently before starting neoadjuvant chemotherapy. We analysed the outcome of patients who had chemo-PORTs with breast surgery for their complication rate to discern the safety of this additional procedure which is meant to improve quality of life. We also want to highlight the 3 major complications we encountered during the study period with an aim to provide effective management of these complications.

## Methods

We analysed a prospective database of breast cancer patients who had concurrent chemo-PORT placement with breast surgery in the non-metastatic setting, between January 2019 and December 2023 at our tertiary cancer centre. All patients had histologically proven breast cancer and underwent breast conservation surgery or modified radical mastectomy with or without reconstruction, either in per primum or post-chemotherapy setting. The surgical and adjuvant treatment related decisions were as per standard institutional practises.

## Procedure

All patients had PORT placement under general anaesthesia. They were prepared with a slight neck extension and the side contralateral to breast surgery was usually preferred (whenever available) for chemo-PORT placement. Chemo-PORTs were inserted using the internal jugular vein (IJV) access. The standard Seldinger technique was applied, either by blind anatomical landmark guidance or ultrasound (USG) guidance, depending on surgeon preference. For blind IJV cannulation, the anatomical landmarks included the triangle formed by the two heads of the sternocleidomastoid muscle and the clavicle, with the puncture site typically at the apex of this triangle just lateral to the carotid pulse, needle directed towards the ipsilateral nipple. Ports were secured in a subcutaneous pocket, which is 2 cm below clavicle on the lateral aspect more laterally to ensure concealment under women’s clothing and to avoid the medial chest region, which is prone to keloid formation. The catheter tip position confirmed with intra-operative or post-operative imaging. Chemo-PORTs were flushed on table with 30 ml (1U in 10 ml) heparinised solution, on post-operative day-1, before chemotherapy and when not in regular use were flushed with every 4 weeks with the same regimen to prevent occlusion.All were pre-connected implantable devices, 9.8 Fr single lumen catheters. The preferred venous access route was internal jugular vein. All patients had an intra-operative and/or post-operative chest X-ray for confirmation of catheter tip. All PORTs were removed within 1 month to 2 years of completion of adjuvant treatment, as per disease stage and patient convenience. The clinico-pathological and demographic data was collected from electronic medical records and analysed in SPSS-V25.

## Results

### Demographic Features

A total of 408 patients underwent chemo-PORT placement during study period. The median age of the cohort was 50 years(18–78). Breast conservation surgery was done in 201(49.3%) patients and 207(51.7%) patients underwent mastectomy. Of 408, 195(47.8%) underwent per primum surgery, 126(30.9%) were operated post-chemotherapy whereas 26(6.4%) were operated for an in-breast tumour recurrence(IBTR) and 61(15%) for a second/contralateral breast cancer. Of the 408, 260(63.7%) patients were HER2 positive, 75(18.4%) were triple negative and 73(17.9%) were hormone receptor positive. A total of 256(62.7%) patients received maintenance trastuzumab therapy for up to one year. All patients had an internal jugular vein(IJV) access and 67% were inserted on the right and 33% on left side. The details of other clinico-pathological characteristics are provided in Table 1.

**Table 1:** Clinico-pathological characteristics of 408 patients with chemo-PORT placement

Clinico-pathological characteristic		N = 408	%
Age	Median	50	
	Mean	49.88	
	Range	18–78	
Gender	Male	0	
	Female	408	
Surgery setting	Upfront	195	47.8
	Post-chemotherapy	126	30.9
	In breast tumour recurrence	26	6.4
	Contralateral breast second primary	61	15
Molecular subtype	HER2neu positive	260	63.7
	Triple negative	75	18.4
	ER/PgR positive	73	17.9
PORT location (IJV)	Right	253	62.01
	Left	126	30.89
	Not known	29	7.10
Surgery type	Breast conservation surgery	201	49.3
	Mastectomy	207	50.7

### Complications

**Fig. 1:**
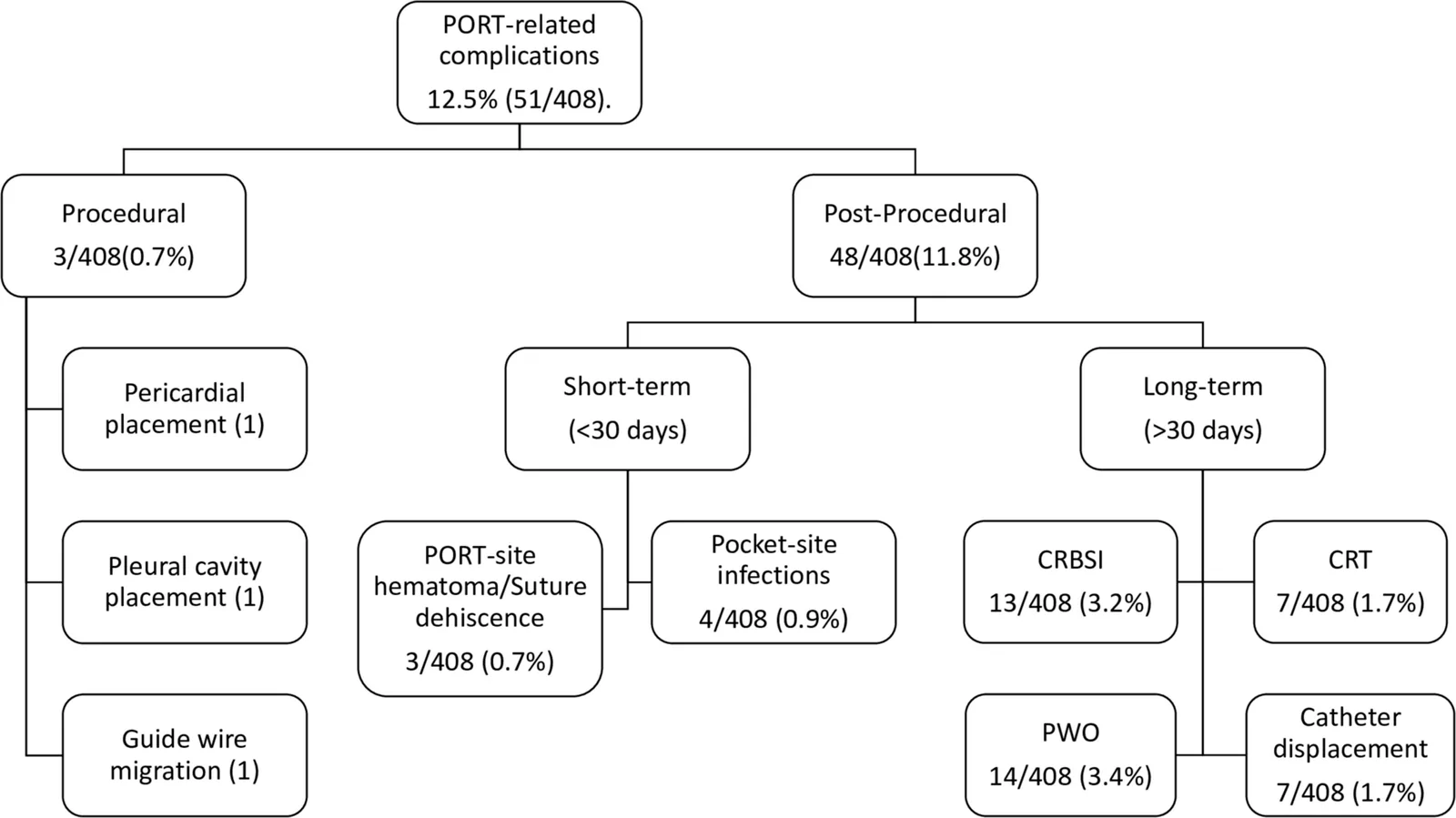
PORT related complications. CRBSI- catheter-related bloodstream infections; CRT-catheter-related thrombosis; PWO- persistent withdrawal occlusion

The overall complication rate was 12.5% (51/408) (Fig-1, Table-2). We found a higher complication rate with left sided (18.2%) as compared to right sided (9.8%) PORT placement (p 0.02).

**Table 2:** Complications of chemo-PORT along with their management

Category	Complication	N (%)	Management
Procedural/Immediate	Pericardial placement	1 (0.2%)	Repositioned and salvaged under Interventional Radiological guidance
	Pleural placement with haemothorax	1 (0.2%)	PORT removed with balloon occlusion; intercostal drainage and transfusion given
	Guidewire migration	1 (0.2%)	Retrieved with snare catheter; PORT salvaged
Post-Procedural: Short-Term (<30days)	PORT pocket-site infection	4 (0.9%)	Antibiotics and Salvaged
	PORT-site hematoma/Suture dehiscence	3 (0.7%)	Salvaged
Post-Procedural: Long-Term	Catheter-related bloodstream infections (CRBSI)	13 (3.2%)	Antibiotics and PORT removal subsequently if infection does not subside and symptoms persist
	Persistent withdrawal occlusion	14 (3.4%)	Observed with continued chemotherapy administration
	Thrombosis	7 (1.7%)	Anticoagulation and subsequent PORT removal
	Catheter displacement (tip or hub)	7 (1.7%)	Repositioned or removed

Post-procedural complications were sub-classified as short-term and long-term complications. Short-term complications included PORT-site hematoma or suture dehiscence in 3/408 (0.7%) and pocket-site infections in 4/408 (0.9%). Long-term complications included catheter-related bloodstream infections in 13/408 (3.2%), catheter-related thrombosis in 7/408 (1.7%), persistent withdrawal occlusion in 14/408 (3.4%), and catheter displacement in 7/408 (1.7%). We classified the procedure-related life-threatening complications as major which were seen in 3/408(0.7%) patients- pericardial placement in one, pleural cavity placement leading to haemothorax in other and intra-operative guidewire migration in one. Interventional radiology support was sought to manage the complications, and all were salvaged successfully.

### PORT Site Infection

**Fig. 2:**
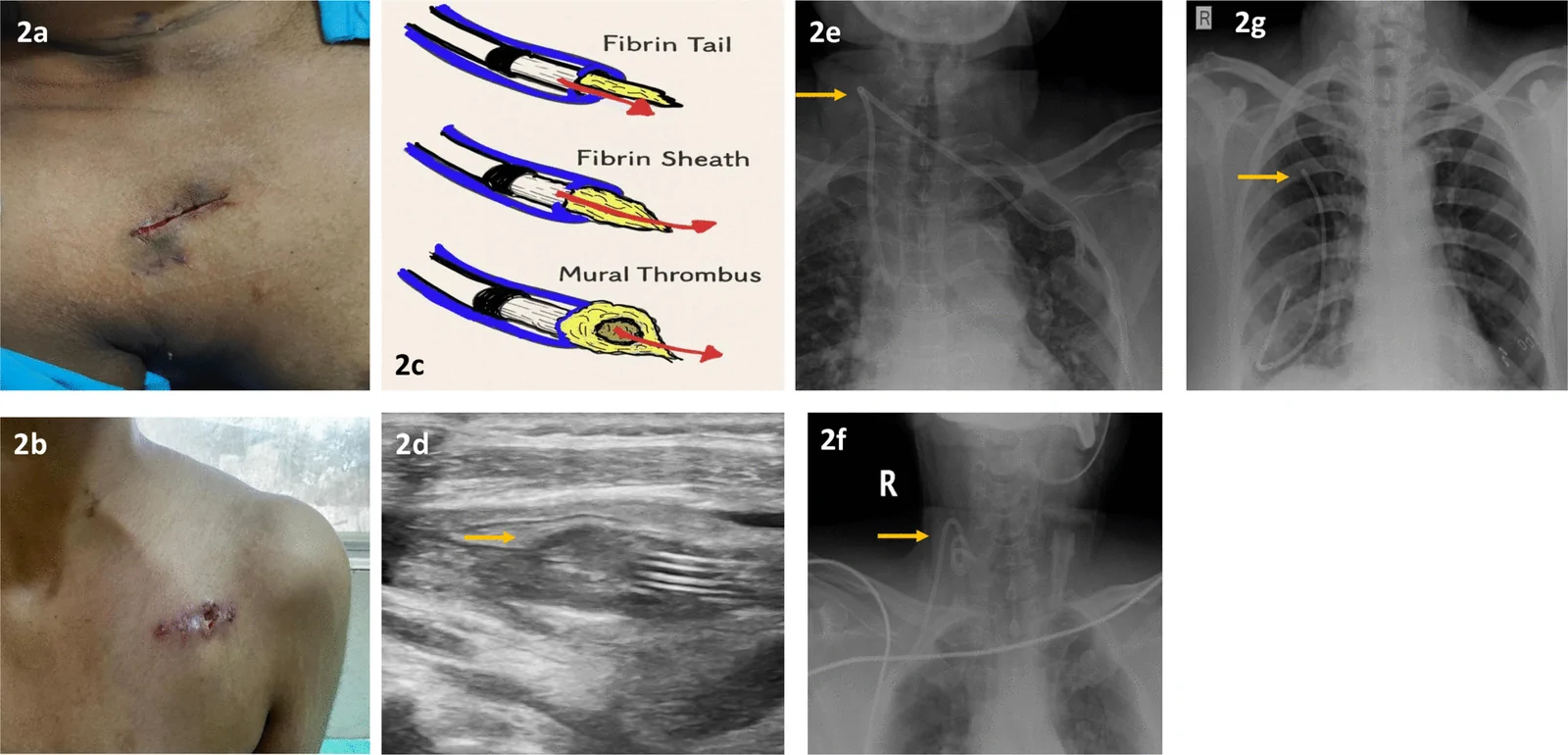
**Minor Complications**. 2a- Superficial wound infection and hematoma, 2b- PORT site wound gape, 2c- Schematic representation of backflow obstruction- due to fibrin tail, fibrin glue and thrombus, 2d- Ultrasound image showing near complete lumen occluding thrombus seen in right IJV for length of 4.7 cm, 2e- PORT hub site kink causing backflow issues, 2f- Recoiling of PORT catheter in the right IJV, 2 g- Recoiling of the PORT catheter in the subcutaneous tissue of chest wall

There were 13 patients who had fever after flushing of PORT and were diagnosed with catheter related blood stream infections (CRBSI). Of these patients, 9 patients had organism detected in the PORT tip cultures—the most common organism isolated was gram negative bacilli (GNB—Kliebshiella, E. cloacea). Four patients presented with local PORT site infection as localised swelling and erythema. Most common organism isolated from PORT pocket infection was gram positive cocci—Staphylococcus aureus (Fig-2a, 2b).

### PORT Catheter Related Thrombosis

Chemo-PORT thrombosis was seen in 7 patients(1.7%). Most of these patients were asymptomatic and detected on routine imaging or due to backflow obstruction. They were managed with anticoagulation—heparin therapy for 3–6 months. All PORTs were removed 3 months post anticoagulation therapy (Fig-2d).

### Catheter Displacement

Kinking of catheter (Fig-2e) at the PORT hub was seen in one post-surgery chest X-ray, which was repositioned under local anaesthesia. One patient had the PORT catheter coiled in the right IJV (Fig-2f) and another had it migrated in the chest wall subcutaneous plane (Fig-2g). Both these could not be salvaged. Displacement of huber’s needle(2) due to deep placement of the PORT hub making it difficult to localized by daycare nurse is possible, especially in overweight patients.

### Pericardial Placement

**Fig. 3:**
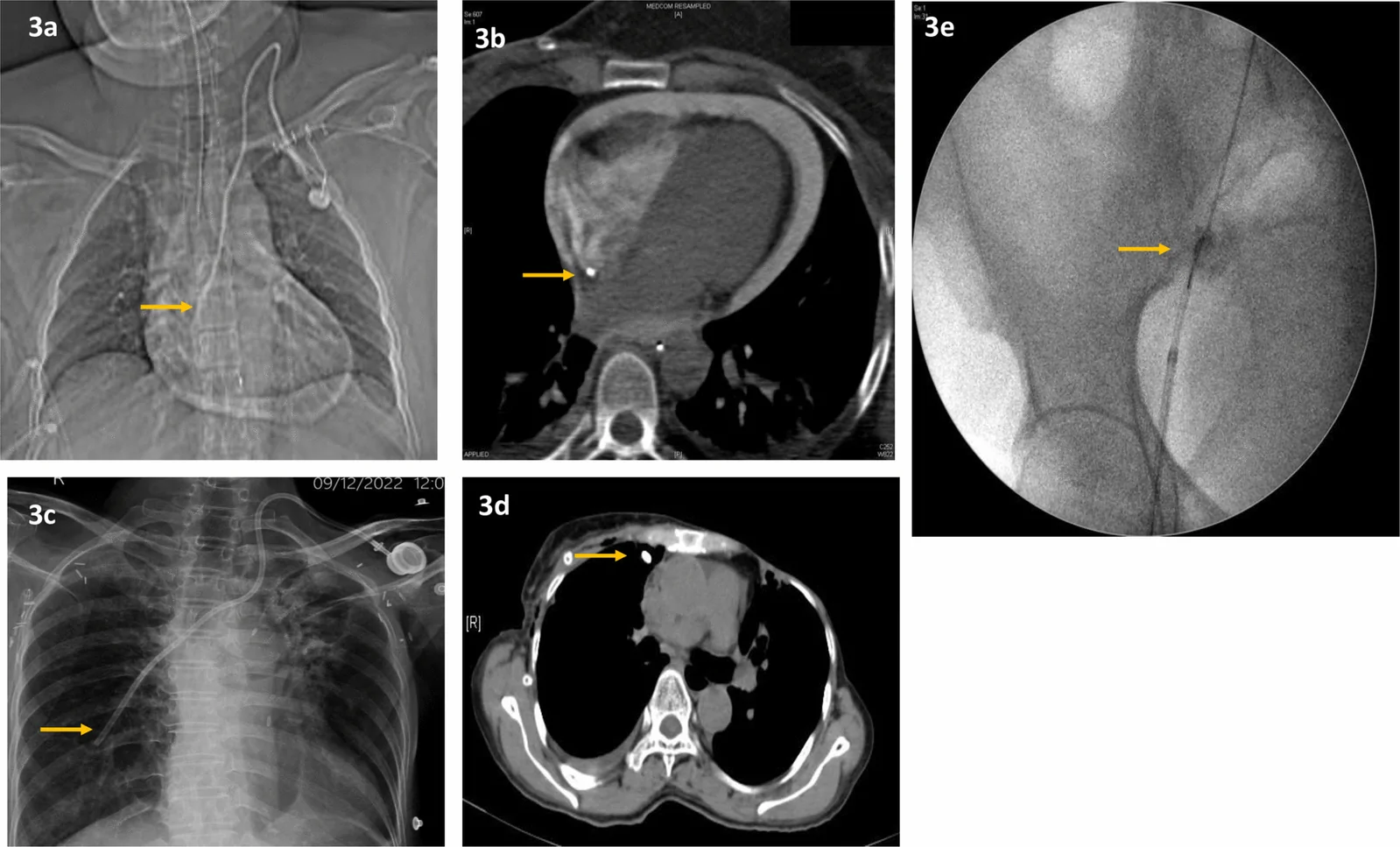
**Major Complications.** 3a- Portogram suggestive of PORT tip in the pericardium, 3b- CT portogram showing dye in the pericardial cavity, 3c- Chest X-ray suggestive of pleural placement, 3d- CT thorax sagittal view confirming PORT tip in the pleural cavity, 3e- Fluoroscopic image showing snare retrieval of guide wire from the external iliac vein

A post procedure C-arm image suggested that the PORT had traversed left innominate vein reaching pericardium (Fig-3a). An intra-operative computed tomography(CT) was performed in the interventional radiology(IR) suite to assess the site of venous injury. The junction of the internal jugular vein(IJV) and brachiocephalic with a high riding pericardium was identified as the site (Fig-3b). Femoral access was secured. Fluoroscopy was performed with a snare and wire introduced using bilateral femoral venous access. The PORT tip was repositioned and secured intravascularly. Angiogram done identified leak for which balloon tamponade was done. The patient recovered uneventfully, PORT was salvaged, and was used subsequently.

### Pleural Placement

During left sided chemo-PORT placement, at the end of routine procedure, there was no backflow and intra-operative C-arm suggested suspicious placement of the catheter into false passage in the pleura (Fig-3c). A post-operative contrast-enhanced CT thorax showed an inadvertent perforation at superior vena cava-brachiocephalic junction and the tip of catheter was in pleural space (Fig-3d). There was no active bleed into the hemithorax. The decision was made to remove the PORT in the IR suite. A median sternotomy and/or thoracotomy was available should the need to suture the venous rent arise. The IR team secured venous access via retrograde femoral vein route under general anaesthesia and chemo-PORT was removed with balloon occlusion of the SVC rent. The patient had 600 ml haemothorax during the procedure which was managed by intercostal drainage (ICD). Patient was transfused blood, observed in ICU for 48 h and she recovered uneventfully. A repeat CT was done to confirm no venous leak, and the ICD was subsequently removed.

### Guidewire Migration

During routine procedure, the guide wire inadvertently slipped into the venous system. An intra operative fluoroscopy was performed by IR team to localise the migrated guide wire in external iliac vein (Fig-3e). A femoral access was secured. A snare catheter- thin flexible catheter with a small basket at its tip was snagged around the migrated guidewire and retrieved under fluoroscopic guidance by the IR team. The PORT was salvaged.

## Discussion

We report an overall 12.5% complication rate during chemo-PORT placement. The incidence of major complication was 0.7%. However, all the 3 major complications were promptly detected and efficiently managed. The most common complication in our series was catheter related infection(4.2%). In a previous systemic review, the complication rate was similar. They reported an infection rate of 6.0% with PICC and 2.1% with chemo-PORTs, thrombotic event 8.9% with PICC and 2.6% with chemo-PORTs [8]. Narducci et al. reported a 16.1% complication rate and infection at 5.3%, accounting for half of premature device removals [9]. PORT related infection includes both the PORT pocket site infection and CRBSI. Factors influencing this include heavy microbial colonization of insertion site, neutropenia, and duration of device usage [10]. Patients presented with redness, inflammation, and pain at PORT site in case of pocket infection whereas CRBSI is diagnosed when patients present with fever and chills on flushing of PORT and/or during chemotherapy. Blood cultures from PORT should be obtained in these cases. Most isolated organism from PORT pocket site infection was gram positive bacteria, Staphylococcus aureus and in CRBSI, gram-negative bacteria namely Pseudomonas aeruginosa, Escherichia coli and the resistant Stenotrophomonas maltophilia, were identified. Pocket infections were treated with antibiotics; PORTs were salvaged if symptoms resolved. In our series all CRBSI cases required removal. Antibiotic lock therapy(ALT) protocol for implanted subcutaneous PORTs is implemented [11]. This involves instilling antibiotics directly into the chemo-PORT and allowing it to dwell. Chemo-PORTs are then flushed, culture repeated 4/5 days after instillation. PORTs may be used again if asymptomatic and if negative culture is obtained. Preventing chemo-PORT infection is critical to ensure the safety and well-being of patients undergoing chemotherapy [12]. Sterile insertion, antimicrobial prophylaxis, maintaining rigorous catheter care, including regular cleaning with antiseptics before each use and timely dressing changes using sterile technique are important in reducing infection rate. One must educate patients and caregivers about maintaining hygiene around the chemo-PORT site and recognizing early signs of infection for prompt reporting.

Persistent withdrawal occlusion(PWO) occurs in case of inability to withdraw blood and infusion of fluids may or may not be smooth [13]. Fibrin tail, sheath and mural thrombus are reasons for withdrawal occlusion. The commonest cause is formation of fibrin sheath which creates a one-way valve, with negative pressure on aspiration causing partial obstruction that is relieved on flushing or infusion. Use of thrombolytic agents is suggested in this situation to restore catheter patency [14]. We encountered this complication in 3.8% patients. Tchirhart et al. demonstrated PWO in 19% patients [13]. All of them had backflow issues, however forward flow was good in most of them. Thrombotic event must be ruled out. PWO is sometimes due to catheter tip abutment at the vessel wall. In the event of a suspected fibrin glue/plug, 10 ml solution of Streptokinase(containing 500 IU/ml) is instilled in the blocked lumen of the PORT catheter slowly by aspiration push method. It is aspirated after 24 h and discarded along with 5–6 ml of blood. The catheter is then flushed with 30 ml NS followed by 10 ml of heparin solution(10 IU/ml). Similar treatment of PWO with 250,000 units of urokinase dissolved in 150 cc D/W infused through the PORT over 90 min is reported [13]. In persistent PWO, we obtained a PORT-ogram, dye study after injection of CT contrast into the chemo-PORT and visualizing the contrast flow through the vessel and heart under fluoroscopy. If the PORT-ogram confirms the entry of contrast into heart chamber and catheter tip is at desired place, then the chemo-PORT use may be continued. However, day care nurses need to be primed regarding the situation.

Another common and serious complication of chemo-PORTs is catheter related thrombosis(CRT). We observed 1.7% incidence of CRT in our cohort. Reducing the risk of thrombosis necessitates both patient education and health care provider training. Cancer itself is a pro-thrombotic condition, and venous thromboembolisms negatively impacts cancer survival, hence, it is crucial to reduce its risk [15]. Several factors contribute to increased risk of CRT. Firstly, foreign body reaction, the presence of chemo-PORT catheter itself may irritate the vein wall, triggering an inflammatory response that can lead to clot formation [16]. Reduced blood flow through the catheter due to infrequent use or improper flushing techniques allows blood to pool and clot, leading to blood flow stasis [17]. Underlying medical conditions such as hypercoagulable states due to inherited conditions or acquired factors like cancer itself or hormonal therapies elevate the risk [18, 19]. Certain catheter materials or designs might be more prone to clot formation, although research is ongoing in this area [20]. Also, infection at the insertion site can trigger inflammation and increase the risk of thrombosis [21]. Most of these patients were asymptomatic and thrombosis was detected when there was no backflow during PORT flushing or on routine imaging for disease. Doppler ultrasound should be carried out if thrombosis is suspected (sensitivity 56%–100%; specificity 94%–100%) [22]. If the ultrasound is normal, and thrombosis is still suspected in the event of PWO, a venography or alternative imaging like CT or MRI should be carried out [23–25]. Patients with CRT are managed with anticoagulation therapy with low molecular weight heparin (LMWH; enoxaparin 0.6 mg subcutaneously twice daily and follow-up imaging. On resolution of the thrombus, PORT was removed in all cases. Some guidelines support continued use of the PORT after anti-coagulation therapy and resolution of thrombus [22]. The primary purpose of removing the PORT is to reduce the risk of pulmonary embolism(PE) [26]. Optimal placement of the catheter tip at the junction of superior vena cava and right atrium without abutting the venous wall, and appropriate use of heparin or saline flush at the end of usage are important factors affecting the incidence of CRT. A meta‐analysis of 5,636 adult cancer patients fitted with central venous catheters(CVCs) showed that, with respect to risk factors for CRT during catheter insertion, implanted PORTs performed better than external catheters, and implantation in the jugular vein was better than that in the subclavian. This meta‐analysis also confirmed that the rate of thrombosis was higher when the CVC tip was located above the junction between the superior vena cava and the right atrium [27]. Anticoagulation therapy should be administered in the event of CRT as there is risk of clot dislodgement and potentially life-threatening complications like PE. Individuals with underlying hypercoagulable conditions might benefit from long-term anticoagulation to prevent future clots [28]. We summarise the various guidelines recommended for management of CRT in Table 3. A recently published Cochrane review addresses the efficacy of thromboprophylaxis in cancer patients with CVC, however the evidence for this was moderate-certainty and there was a potential threat of haemorrhagic complications with anti-coagulants [29].We do not use thromboprophylaxis routinely in this situation, unless for treatment of CRT.

**Table 3:** Guidelines recommendations for the treatment of catheter-related thrombosis^22^

	NCCN	ASCO	ACCP	ESMO	International Society on Thrombosis and Hemostasis
Treatment	No specific drug recommendation	LMWH	UFH, LMWH or fondaparinux rather than, vitamin K antagonist (VKA)	LMWH, VKAs or LMWH followed by VKAs for 3–6 months	LMWH. Oral VKA can also be used, in the absence of direct comparisons of these two types of anticoagulants in this setting
Duration	At least for 3 months or as long as the catheter is in place	Extended therapy with LMWH or VKA beyond 6 months if: Metastatic disease Active chemotherapy Recurrent thrombosis	Extended therapy is preferred to 3 months of treatment	As long as the catheter is in place and functional	3 months
Catheter removal	If thrombosis symptoms persists, infection, dysfunctional line or when the line is no longer needed	Not described	If unfavourable clinical evolution under anticoagulation	If non functional concomitant deep vein thrombosis, sepsis. If CVC is no longer needed or long-term anticoagulation is contraindicated, a short course of 3–5 days is advised	Need not be removed if functional, well‐positioned and non‐infected with good resolution of symptoms under close surveillance

*NCCN* National Comprehensive Cancer Network; *ACCP* American College of Chest Physicians; *ASCO* American Society of Clinical Oncology; *ESMO* European Society for Medical Oncology

Displacement of catheter can be both an intra-operative and late complication. Displacement occurred in 10 out of 408 patients(2.5%) in our series, of these 3 patients had intra-operative malposition leading to adverse complications, all of which were managed promptly. The other 7 had catheter tip displacements in the IJV(3), subcutaneous in chest wall(2) and displacements of the PORT hub(2). Displacement of the Huber’s needle was observed in 2 patients, mainly due to deep PORT hub placement, a problem accentuated in overweight patients. Preventive measures include optimal subcutaneous depth during insertion, accurate pocket marking, and nursing staff training in localisation techniques to ensure safe and reliable access. Intra-operative confirmation of catheter tip position using on table fluoroscopy with C-arm machine is useful, however not mandatory. A retrospective study compared 107 chemo-PORT placements without fluoroscopy and reported no difference in complication rate with or without intra-operative fluoroscopy. Fluoroscopy is an expensive imaging technique and not universally available, eliminating its use resulted in cost savings of approximately $40,000 [30]. Hence, appropriate technique and judgement of catheter length can be a reasonably good guide in the absence of intra-operative fluoroscopy. Other studies have also shown that routine chest X-ray after fluoroscopy guided totally implantable venous access device insertion is not mandatory [31]. Gebauer B et al. reported using a combination of ultrasound and fluoroscopy guidance for PORT a well-established approach known for its high success rate and low complication profile [32]. The complications due to chemo-PORT catheter tip displacement can occur with long standing PORTs. An unusual case of chemo-PORT catheter tip migrating from the heart to the axillary vein in a patient with severe cough has been reported [33]. The catheter was repositioned using a minimally invasive procedure, trans-femoral snaring.

There are several reports of major complications during chemo-PORT placement. Biswas et al. report a mediastinal hematoma resulting from vascular injury during chemo-PORT placement. The patient was managed by urgent thoracotomy [34]. Subclavian catheterization is more likely than internal jugular catheterization to be complicated by pneumothorax and hemothorax [35]. Chemo-PORT pleural placement leading to hemothorax was managed with a video-assisted thoracoscopic surgery for evacuation of the hemothorax [36]. We managed most major complications with the help of our IR team and hence major surgery like sternotomy or thoracotomy could be avoided in all 3.

We reported a higher complication rate of left sided PORTs(18.2%) as compared to right(9.8%). This could be due to difference in anatomy of venous system [37] and a relative ease of access for right-handed operator [38]. There is higher chance of endothelial damage due to the catheter's downward thrust. In addition to predisposing to infection, risk of CRT also increases due to chronic microtrauma generated by mechanical stress and endothelial damage sue to toxicity of chemotherapy drugs. The right IJV hence is a more suitable site for PORT insertion since it travels a relatively straighter path to superior vena cava. Ignatov et al. also similarly reported higher complication with left sided PORTs [39]. However, a few others refuted such laterality correlation [12, 40].

The strength of our study lies in the large sample size from a single institution, reflecting a real-world experience of chemo-PORT placements in a diverse cohort of breast cancer patients undergoing various treatment strategies. The limitations include the retrospective nature of the study, lack of comparison with the other type of chemo-PORTs available and inability to capture the patient related outcomes with respect to satisfaction and quality of life with chemo-PORTs.

## Conclusion

We report a complication rate of 12.5% of chemo-PORT placement with 0.7% life-threatening or major complications. One needs to be vigilant at every step and follow a standard operating procedure to reduce major intra-operative complication rate. Prompt detection of serious side-effects and interventional radiology support for managing complication can avoid morbidity as well as mortality.
